# Carbolic Acid in Mammary Abscess, Hypodermically

**Published:** 1889-07

**Authors:** D. B. McGee

**Affiliations:** May, Texas


For Daniel's Texas Medical Journal.

### CARBOLIC ACID IN MAMMARY ABSCESS—HYPODERMICALLY.

BY D. B. M'GEE, M. D., MAY, TEXAS.

IF YOU will be indulgent enough to allow me space in your valuable journal, I will attempt to give a brief synopsis of two cases of mammary abscess treated with hypodermic injections of carbolic acid.

CASE 1—Mrs. B., age 31 years, was confined May 20 of current year, at which time I delivered her of twins. Nothwithstanding her previous health at a low ebb, she hadn't an untoward symptom, save slight adhesions of one placenta, until the 12th day, when her husband informed me that her nipple on right mamma was very "sore," for which I sent the usual remedies. In about four days subsequent to that time, he again came and said he would like for me to visit her. I found her in the following condition, to-wit: Temperature slightly elevated; tongue coated, with white fur, bowels constipated, appetite good; right mamma considerably enlarged, very much indurated, tender and very painful. I ordered saline purge, and injected deep into the gland carbolic acid gtt, ii, with morphia et atropia sulphates ¼ et 1.150 grs., respectively, in ℨij of water, which relieved all pain and prevented formation of pus, as the tumefaction and induration both subsided within a day or so, and she is now well.

CASE 2—Mrs. K., aged 38, was confined March 2, this year; subsequently developed a mammary abscess, from irritation of tight clothing, which had advanced to the formation of pus. Consulted me April 4, at which time I advised an operation; but she couldn't "bear the knife," so I thought it expedient to try the acid carbolic injection on her, and was again rewarded by the subsidence of all symptoms and the absorption of the pus already formed. I have purposely omitted the pathology of mammary abscess and the "physiological action" of carbolic acid, as I trust we are all too familiar with same for it to be worthy of space here. The indication being its antiseptic property to arrest the formation of pus. Have also used it successfully many times in absorbing furuncles, etc., hypodermically.

I hope to hear from other brothers on the subject. Long may the JOURNAL live!

